# How to Find the Drug

**Published:** 1888-12

**Authors:** N. W. Vandenburg

**Affiliations:** Fort Edward, N. Y.


### HOW TO FIND THE DRUG.

N. W. Vandenburg, A. M., M. D., Fort Edward, N. Y.

In an article in the September number, by the editor, we are shown how this is done so well, that any attempt to add to it may seem superfluous.

In quoting Hahnemann the writer says: "Each medicine produces particular effects in the body of man, and no other medicinal substance can create any that are precisely similar."

We may characterize "these peculiar effects" perhaps as well by the term "*individuality*" of the drug as by any way, understanding by that term "the particular effects which no other medicinal substance can create *precisely similar*."

Did we wish to question the universality of this proposition, a doubt might remain in definitions. If by "medicine" and "medicinal substance," different expressions evidently intended for the same thing, Hahnemann meant different *drug-plants*, that is one thing; if different *chemical substances*, that is another. It is well known that many different plants contain the same chemical substances (compounds), and hence may produce the same results. But with Hahnemann's instinctive German liking for fine distinctions, it is fair to infer he meant different drug-plants.

Now it is true that to the last extreme of fineness there may be a lacking of "precisely similar" effects.

But it is also true in the "art of prescribing," that Pulsatilla nuttalliana (really now, Anemone potens L., ver. nuttalliana) has for a long time been used in this country in the place of Pulsatilla nigra of Europe, and not a few prescribe Veratrum viride for V. album. In both these cases the results are satisfactory.

As to the Puls., it is labeled Puls. nig., and no one is the wiser.

It may be argued that the chemical substance is the same in each of the related drugs.

But the main question we wish to look at is in what this "individuality" consists.

We are told that it consists in the "*striking, remarkable, uncommon*, and *peculiar*" signs and symptoms.

We are also told that by these are not meant—though they be very strongly marked in the action of the particular drug in question—the symptoms that occur in many [new] drugs in common.

In the further search for "the characteristic symptoms, * * which no other drug produces in a precisely similar manner," we are told "the mental symptoms are the most important," because "they are more indicative of the *individuality* of the patient than the physical symptoms."

Are we to understand, then, that we are to give *mental symptoms* the preponderance in deciding upon the remedy? So it would seem; but it is not so, as we shall see further on.

"It is the *totality of the peculiar and uncommon symptoms* of the patient that must be covered by the *characteristics of a drug*."

Thus far we have been too indefinitely definite. Once more we read, "A symptom may be *very peculiar* (*or uncommon*), as a *concomitant* to one disease and with another not at all so." Then it is not the *symptom* that is peculiar, but the *concomitance*.

Thus, for example, "profuse micturition" is not a peculiar or uncommon symptom; but "profuse micturition relieving headache" becomes a *peculiar concomitance*.

"So many common symptoms may be qualified or defined so as to make it a peculiar or characteristic one," BY ITS CONCOMITANCE.

This is what the above example, together with others given, seems to mean, if it mean anything.

The same thing is true in the example quoted of Dr. Guernsey. "He did not pick out one or two symptoms and give a drug for these only."

In the case quoted from Hahnemann, we have seven symptoms given, and Bryonia is prescribed "in a low potency, one dose, and the patient was able to work the next day," because, in the comparison with other drugs, it produces the remaining symptoms in a very similar manner" to the disease, which the other drugs do not do; hence Bryonia is to be preferred.

So, too, in the case following, it is the *concomitance* that determines. Hahnemann calls it the "totality of symptoms," and this phrase is always on his tongue. "Totality of symptoms" has with some come to mean *one striking and peculiar* symptom; with others it has come to be "a tripod" of three on which to base the prescription.

With others it is the "exclusion of the general and indefinite" on general principles.

One more quotation of a quotation from Hahnemann, and in it we wish to change the italics so as to logically agree with what has been said above:

"The general and indefinite, such as loss of appetite, headache, weakness, restless sleep, discomforts, etc., *if they are not more closely defined*, deserve little attention, for one finds something about as *indefinite* in almost every sickness, and caused by almost every drug."

How they may be *more closely defined* has already been shown.

"A symptom may be *very peculiar* as a *concomitant* to one disease and with another not at all so."

"*Each drug has its particular symptoms, which, taken collectively, surely indicate that drug.*" Why? Because of their *concomitance.*

This is the great and important sentence in Dr. Lee's article, and not the one he emphasizes.

The bane of homœopathic prescribing has been the looking for "*peculiar and uncommon symptoms* exhibited by the patient" and then finding that drug whose "particular effects" are most similar, and leaving out the word *totality*, as I have done, in quoting Dr. Lee in this sentence.

*Concomitance* is the word, or *totality* if you prefer, not *peculiar or uncommon*, as emphasized by Dr. Lee.

In this looking for the *peculiar and uncommon*, the large bulk of homœopathic prescribers have forgotten the "weightier matters of the law." They have become blinded by the glamour of *peculiar and uncommon;* they examine the patient and scrutinize each symptom manifested to hit upon a *peculiar* and *uncommon* one, and finally they bring up at some trifle that is new or strange to them, and away they go to look for this symptom. If, now, one or two others can be twisted to coincide with the remedy exhibiting this *peculiar and uncommon* one, they seldom hesitate to prescribe that drug.

In how many cases is this true? How many brilliant cases do we not see reported on one or two *peculiar* and *uncommon symptoms?* And how many failures do we not hear of because based upon a tripod of whimsical, peculiar, and uncommon symptoms?

In the example quoted from Hahnemann, not one of the seven symptoms given are *peculiar or uncommon.* On the contrary, they are each common to several or more drugs. The *concomitance in one drug* is peculiar and uncommon, and that is the reason why Bryonia cured. Any other drug that could exhibit a like *concomitance* would have cured as well.

The sooner we return to the "*totality* of all the symptoms manifested by the patient," the more wonderful will be our success.

The words *peculiar and uncommon* will not guide us to this end if they are made the *pole-star* of our investigations.

If we are constantly on the lookout for *concomitance,* if this is the ever-present thought in patient-study and drug-study, then we shall arrive at "the whole art of prescribing, which consists in finding for each patient that drug whose (leave out the word "*particular*") effects are most similar to the *totality* (leave out "the peculiar and uncommon," they are misleading) of symptoms exhibited by the patient."

The *peculiar* and *uncommon* do not consist in the symptoms, but in the CONCOMITANCE.

Now, it is this very concomitance that is the hardest to arrive at from our present materia medica. Hahnemann's arrangement absolutely destroys this relation of concomitance. Instead of getting a clear picture of the drug-sickness as actually caused by that drug, the beginning is placed at the end, the middle is at the beginning or anywhere else, and the end comes first or middle or wherever chance falls.

With all stages thus confused, no wonder that concomitance has become a bore and totality a myth. Few can carry more than the merest outline of a drug until after years of study.

That prescriber is rare who has more drugs at his fingers' ends than he has fingers.

What shall we do to enter the kingdom of materia medica?

